# Acute kidney injury post-abdominal surgery in infants: implications for prevention and management

**DOI:** 10.3389/fped.2023.1162863

**Published:** 2023-04-21

**Authors:** Minh Dien Duong, Silvia Kwak, Naina Bagrodia, Abby Basalely

**Affiliations:** ^1^ Pediatric Nephrology, Norton Children's Hospital, University of Louisville, School of Medicine, Louisville, KY, United States; ^2^ Pediatric Nephrology, Cohen Children's Medical Center of New York, Zucker School of Medicine at Hofstra/Northwell, New Hyde Park, NY, United States; ^3^ Pediatric Surgery, Cohen Children's Medical Center of New York, Zucker School of Medicine at Hofstra/Northwell, New Hyde Park, NY, United States

**Keywords:** postoperative management, neonatal AKI, AKI (acute kidney injury), abdominal surgeries, preoperative consideration

## Abstract

Acute kidney injury (AKI) is common in critically ill infants and is associated with long-term sequelae including hypertension and chronic kidney disease. The etiology of AKI in infants is multifactorial. There is robust literature highlighting the risk of AKI after cardiothoracic surgery in infants. However, risk factors and outcomes for AKI in infants after abdominal surgery remains limited. This article reviews the epidemiology and association of abdominal surgery with postoperative AKI and suggests methods for AKI management and prevention. Postoperative AKI may result from hemodynamic shifts, hypoxia, exposure to nephrotoxic medications, and inflammation. Infants in the intensive care unit after intraabdominal surgeries have a unique set of risk factors that predispose them to AKI development. Prematurity, sepsis, prolonged operation time, emergent nature of the procedure, and diagnosis of necrotizing enterocolitis increase risk of AKI after intrabdominal surgeries. Prevention, early diagnosis, and management of AKI post-abdominal surgery is imperative to clinical practice. Close monitoring of urine output, serum creatinine, and fluid status is necessary in infants after abdominal surgery. A recent study suggests elevated levels of a urinary biomarker, neutrophil gelatinase-associated lipocalin (NGAL), 24 h after an abdominal procedure may improve early prediction of AKI. Identification of risk factors, avoidance of nephrotoxic medications, careful fluid balance, early detection of AKI, and maintenance of hemodynamic stability is imperative to potentially prevent and/or mitigate AKI.

## Background

AKI occurs in over a third of critically ill infants in the neonatal intensive care unit (NICU) and is estimated to confer a fourfold risk of mortality (1–3). The sequelae of AKI are not limited to the kidney. Crosstalk between injured kidneys and other organ systems can increase the risks of intraventricular hemorrhage, respiratory failure, and hypertension (4).

Neonatal AKI risk factors include those that are similar to adults and children, such as hemodynamic and fluid shifts, hypotension, exposure to nephrotoxic medications, and sepsis, in addition to unique risk factors for infants that affect the kidneys, such as prematurity, immature nephrons, low nephron number, and low birth weight (3). Surgical interventions are another risk factor for AKI development. The risk factors, associations, and outcomes of AKI post-cardiac surgery in infants are well detailed. However, there is limited data about the epidemiology and outcomes of AKI in infants after abdominal surgery.

Surgical intervention for abdominal pathology is common with major congenital anomalies present in 2%–3% of infants and necrotizing enterocolitis (NEC) occurring in 1 out of 1,000 infants. Postoperative mortality in infants undergoing abdominal surgery is estimated to be as high as 30% and may be exacerbated by AKI development (5–7). Risk factors for postoperative AKI include (1) preoperative clinical characteristics such as gestational age under 32 weeks, low birth weight, and development of NEC; (2) intraoperative characteristics of hypothermia and longer operative time (>120 min); and (3) postoperative variables such as sepsis and hemorrhage (8–13). Early identification of infants at-risk for postoperative AKI may improve long-term outcomes. Thus, this report aims to summarize and review the epidemiology, risk factors, and outcomes of postoperative neonatal AKI associated with intraabdominal surgery as well as current surveillance and management recommendations.

## Epidemiology of AKI post-abdominal surgery

AKI is estimated to occur in 30% of infants admitted to the NICU (14). Neonatal AKI has been correlated to increased length of mechanical ventilation, hospital length of stay, and mortality (15). AKI after abdominal surgery is estimated to occur in 6.7%–75.3% of infants (Table 1). This wide range of estimates is likely related to varying populations, pathologies, surgical procedures, and AKI definitions. The majority of studies utilize the 2017 modified neonatal Kidney Disease Improving Global Outcomes (KDIGO) creatine and urine output criteria to define AKI (Supplementary Table S1) (3, 10). Postoperative AKI was an independent risk factor for mortality with up to a fourfold increase compared to infants who underwent surgery and did not develop AKI (11, 28).

**Table 1:** Summary of studies postoperative neonatal AKI rates, associated clinical pre, intra, and postoperative variables and mortality

Author	Methodology/ Study Design	Population	Surgical Procedures ^ ^	Surgical Type	Age	AKI Rate for Surgical Procedure		AKI Definition	Clinical Variables associated with AKI	AKI Associated Mortality
						All Stages	Stage 2–3			
Niknafs N et al. (8)	Single-center retrospective study	60	60	Abdominal	0–7d	6.7%	–	SCr increase >1.5 times of baseline or >26 mmol/L, or oliguria (UOP <0 .5mL/kg/h over 24-hour).	Every 1% increase in weight gain within the first 3 days post-op, was associated with 0.6 more days of ventilatory support (p=0.012)	–
Wu Y et al. (9)	Single-center retrospective study	160	41	Thoracic	0d–28d	43.9%	34.1%	Modified KDIGO guidelines SCr/UOP criteria (54, 55)	• Age <32 weeks (aOR 4.8, 95%CI 1.8-12.6) • Necrotizing Enterocolitis (NEC) (aOR 4.3, 95%CI 1.7–11.3) • Sepsis (aOR 3.5, 95% CI 1.3–9.1) • Operation time> 120 minutes (aOR 2.7, 95%CI 1.1–6.6)	aOR 4.3 (95%CI 1.1–16.9)^a^
			119	Abdominal		30.3%	20.2%			
Slagle C.L et al. (12)	Single-center prospective observational study	141	40	Thoracic	0d–6mo	15.1%	–	Neonatal KDIGO guidelines SCr/ UOP criteria (56)	• %Increase in uNGAL pre and 24 hours post operatively predicted AKI development (AUC^1^ 0.81, 95% CI 0.72, 0.89)	Any AKI: aOR 11.1 (95%CI 2.0–62.8)^a^ Severe AKI: 20.3 (95%CI 4.8–85.4)^a^
			86	Abdominal		30.3%	–			
Cui Y et al. (10)	Single-center retrospective case-control study	329	11	Thoracic	0d–28d	36.4%	–	• Modified KDIGO guidelines SCr criteria • Intraoperative oliguria, UOP <0.5 mL/kg/h (15)	• Preoperative mechanical ventilation (aOR 3.47, 95%CI 1.05-11.46) • **Surgical duration, min (aOR 0.98, 95%CI 0.96–0.99), p=0.001**	AKI N=7 (23.3%) vs. No AKI N=30 (10%), p=0.27^a^
			318	Abdominal		8.2%	–			
Cui Y, Cao R, and Deng L (11)	Single-center retrospective study	295	6	Thoracic	0d–28d	33.3%	–	Modified neonatal KDIGO guidelines SCr criteria (57)		aOR 3.49 (95%CI 1.3–9.3)^a^
			292	Abdominal		8.9%	–			
Yum S.K et al. (13)	Single-center retrospective study	53	99	Bedside Abdominal	–	49.5%	–	pRIFLE, AKIn and modified KDIGO (18)	• Preoperative acidosis pH <7.15 or base deficit > 10 (aOR 11.067, 95%CI 2.50–49.02) • ↑ **Preoperative UOP ml/hr (aOR 0.55, 95%CI 0.31–0.96)**	AKI vs. No AKI (26.9% vs 11.1%)^a^
Criss C.N et al. (58)	Single-center retrospective study	181	48	Abdominal	–	62.5%	–	Modified neonatal KDIGO guidelines SCr criteria (15)	–	HR 2.4 (95% CI 1.2–4.8), p = 0.008^b^
Aviles-Otero N et al. (19)	Single-center retrospective study	146	8*5*^c^	Abdominal	0–7d	75.3%^d^	–	Modified neonatal KDIGO guidelines SCr criteria (59)	• **Preoperative exposure to caffeine^c^ (aOR 0.04, 95%CI 0.00–0.37)** • Mechanical ventilation^c^ (aOR 6.09, 95%CI 1.74–21.33) • **Age (weeks)^c^ (aOR 0.77, 95%CI 0.62–0.97)** • **Surgery type: Laparotomy^c^ (aOR 0.11, 95%CI 0.01–0.97)**	aOR 9.49 (95%CI 2.15–41.99)^c^
Garg PM et al. (21)	Single-center retrospective observational cohort study	202	104	Abdominal	–	–	61%	Modified KDIGO guidelines for SCr/ UOP criteria (UOP <1 mL/kg/h over the last 24 h) (3, 14, 15, 57, 60)	• Surgical NEC and severe AKI (aOR 30.68, 95%CI 8.98–130.67)	–
Bakhoum CY et al. (61)	Multicenter retrospective study	77	15	Abdominal	0–1yo	60%	–	Modified KDIGO guidelines SCr criteria (61)		HR 20.3 (95% CI 2.5–162.8)^c^

SCr, Serum Creatinine; UOP, Urine Output; uNGAL, urinary neutrophil gelatinase-associated lipocalin; KDIGO, Kidney Disease Improving Global Outcomes; pRIFLE, Pediatric Risk, Injury, Failure, Loss, End-Stage Renal Disease; AKIN, Acute Kidney Injury Network; aOR, Adjusted Odd Ratio; CI, Confidence Interval; HR, Hazards Ratio.

**Bolded text**: variables protective against AKI development

^a^Rate reported as a combined rate for thoracic and abdominal surgery.

^b^Area under the receiver operator curve.

^c^Combined association of surgical and medical NEC with AKI.

^d^Additional data obtained from author during correspondence.

This review included a single-centered prospective study and nine single-/multicentered retrospective studies evaluating AKI after abdominal surgery in infants up to one year of age. A total of 1,239 procedures and 1,141 intraabdominal procedures were performed (Table 1). The underlying pathology for surgery was categorized by etiology: (1) *Peritonitis* including NEC and gastrointestinal perforation; (2) *Intestinal Obstruction* due to intestinal atresia or stenosis, meconium ileus, and Hirschsprung's disease; (3) *Abdominal Wall Anomalies and Compartment Syndrome* including gastroschisis and omphalocele (Supplementary Table S2) (9–12, 29).

(1) **Peritonitis**

Inflammation of the peritoneum increases risk of bacterial translocation from the peritoneal cavity to the blood thus increasing risk of sepsis (20). The incidence of neonatal AKI in pathologies related to peritoneal inflammation such as surgical NEC, gastrointestinal perforation, and appendicitis are reported to be between 6.7%–75.3% (8–13, 22). NEC and spontaneous intestinal perforation (SIP) are common, affecting the gastrointestinal tract of 5%–7% of infants in the NICU and have a strong association with AKI (30). A retrospective study at the University of Mississippi Medical Center demonstrated that severe AKI occurred in 32.6% (66/202) of neonates diagnosed with NEC (with and without surgery) (24). SIP, although a pathology distinct from NEC, has high rates of postoperative AKI. The reported incidence 41.2% (*n* = 21/51) of infants developed AKI with surgical NEC/SIP (9). Infants with NEC and SIP have multiple risk factors for AKI, including sepsis, hemodynamic instability, postsurgical inflammatory response, increased intraabdominal pressure, and nephrotoxic medication exposure.

Surgical NEC showed stronger association with severe AKI (stage 2–3) compared to medical NEC (aOR 30.68, 95% CI 8.98–130.67) (24). The highest rates of AKI were reported in a retrospective study of 85 infants undergoing peritoneal drainage (*n* = 54, 50%) and/or laparotomy (*n* = 10, 83%) for NEC and SIP in a single center in Virginia (Carmody JB, personal communication December 2022).

(2) **Intestinal Obstruction**

Neonatal intestinal obstruction occurs 1 in 1500–2000 live births and is the most common surgical emergency in the neonatal period (31–33). If diagnosed early, infants with intestinal obstruction rarely have electrolyte imbalance and dehydration, which are common risk factors for AKI (34). Intestinal atresia is the most frequent cause of neonatal intestinal obstruction (35). A single-centered retrospective observational cohort study by Wu and colleagues and a single-centered prospective observational study in Cincinnati reported an AKI rate between 20%–23% in infants with intestinal atresia/stenosis (9). The location and etiology of the obstruction (anal atresia vs. meconium ileus vs. ileal atresia) were not significantly associated with postoperative AKI (9, 11).

(3) **Abdominal Wall Anomalies and Compartment Syndrome**

Gastroschisis and omphalocele are the two of the most common abdominal wall defects in infants and are reported in a large multicenter multinational retrospective cohort of neonatal AKI to date, the AWAKEN study, to occur in 2.5% of infants with AKI (3, 35). Prior to a surgical correction, gastroschisis can lead to excessive insensible fluid losses and thus may further increase the risk of AKI (36). After bowel reduction, the potential for increased intraabdominal pressure and arterial hypotension decreases perfusion pressure within the abdominal arterial vessels and can result in hypoxic renal injury (35, 37).

Animal models of gastroschisis and abdominal compartment syndrome demonstrated higher levels of urinary MCP-1 proinflammatory chemokines and VEGF and π-GST biomarkers of hypoxic kidney damage. These findings were corroborated in newborns with gastroschisis and congenital diaphragmatic hernia, suggesting that these biomarkers may serve as early markers of AKI during periods of intrabdominal hypertension and renal hypoperfusion (38).

## Risk factors for AKI after intraabdominal surgery

Knowledge of renal physiology is imperative to appreciate infants' unique AKI risk factors. Infants are at increased susceptibility for AKI due to: (1) lower renal blood flow, especially during the first week of life, (2) lower glomerular filtration rates, (3) impaired ability of urinary concentration and if preterm or small for gestational age (4) a lower number of nephrons to recover from direct kidney injury (39, 40).

**Figure 1:**
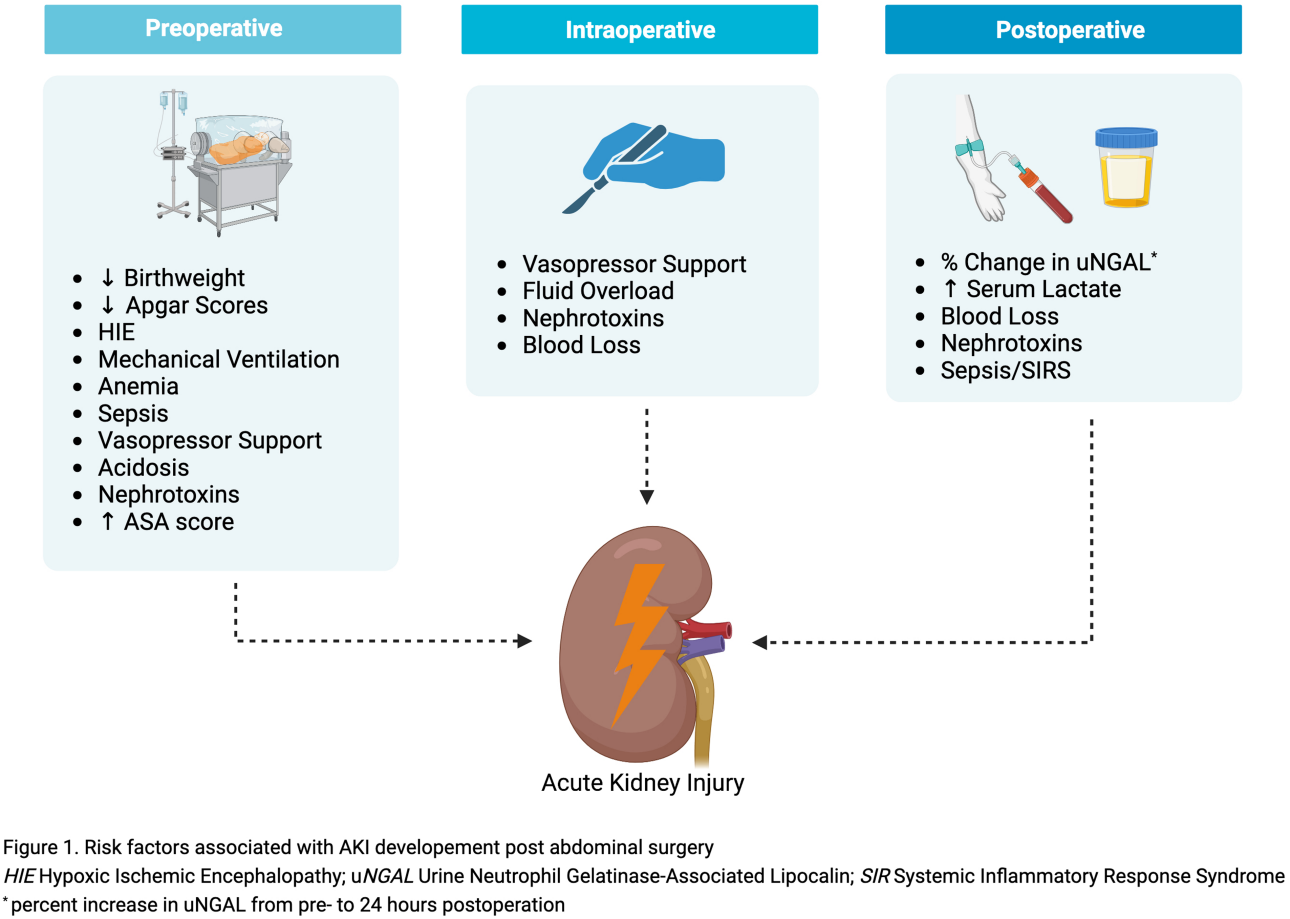
Risk factors associated with AKI developement post abdominal surgery HIE hypoxic ischemic encephalopathy; uNGAL urine neutrophil gelatinase-associated lipocalin; SIR systemic inflammatory response syndrome *percent increase in uNGAL from pre- to 24 h postoperation.

Surgical intervention is an additional risk factor for neonatal AKI (40, 41). Hypoxia, mechanical ventilation, hemodynamic shifts, blood loss with resultant anemia, and exposure to nephrotoxic medications (antibiotics, anesthetics, diuretics) are common prior, during, and after abdominal surgery (Figure 1) (9, 10). Abdominal pathology may also be associated with congenital abnormalities of the kidneys and urinary tract (CAKUT). Infants with CAKUT have increased susceptibility to AKI, in which acute insult can compound kidney dysfunction (42).

### Preoperative risk factors

#### Birthweight, apgar scores, and gestational age (GA)

Prematurity and low birth weight are associated with increased risk of AKI development due to decreased nephron number and maladaptive repair. In addition, immature tubular function limiting urinary concentration leads to increased insensible losses and dehydration, increasing susceptibility to injury (40). In the AWAKEN study AKI developed in 45% of extremely premature infants (<29 weeks GA) as compared to 14% of infants between 29 and 36 weeks GA (3). Studies specifically evaluating AKI post-abdominal surgery demonstrate similar associations of lower birth weight (<1500 grams), gestational age (under 32 weeks), and Apgar scores with increased risk of AKI (10).

#### Respiratory distress and mechanical ventilation

Infants requiring ventilatory support prior to abdominal surgery are at increased risk of postoperative AKI (10, 41). A study of 329 Chinese infants at a single Children's hospital found that of the 44 infants were ventilated preoperatively, a quarter developed AKI postoperatively (*n* = 11). Even after adjusting for other AKI risk factors, infants ventilated preoperatively displayed 3 times higher risk of AKI (aOR 3.47, 95% 1.05–11.46) (10). The association between mechanical ventilation and AKI development may be related to hypotension, decreased kidney perfusion, and potentiated inflammation (6, 9). It has been postulated that higher mean airway pressure during positive pressure ventilation can decrease kidney perfusion (41). In addition, studies over the past three years demonstrate the complex crosstalk between the lungs and kidneys. Lung inflammation can potentiate kidney inflammation through the release of pro-inflammatory cytokines initiating a cascade of immune cell response with molecular products damaging renal tubular cells (43).

#### Systemic inflammation and sepsis

Systemic inflammation and sepsis are common risk factors for neonatal AKI (3). AKI in infants with sepsis is multifactorial, including sepsis-related systemic inflammatory response, hypotension, vasculature micro thrombosis, ischemia and necrosis, and/or nephrotoxin exposure (9, 44). Postoperative sepsis is strongly associated with AKI (9). Wu et al. found sepsis to increase the odd of AKI by over three-fold, even after adjusting models for surgical diagnosis and prematurity (aOR 3.5, 95% CI 1.3–9.1) (9).

#### Acidemia

A pH <7.15 or base deficit >10 prior to abdominal surgery has been shown to be associated with the development of postoperative AKI (45). Metabolic acidosis is in neonates with severe illness and can potentiate inflammation and decrease renal blood flow (45, 46).

#### Nephrotoxic medications

It has been well established that exposure to nephrotoxins increases the risk of AKI. Aminoglycosides (gentamicin), Vancomycin, NSAIDs (Indomethacin), and loop diuretics (furosemide) are common medications utilized prior to and during NICU stay (39, 40, 47). Treatment with vancomycin (OR 1.637, 95% CI 1.175–2.280) and loop diuretics (aOR 2.20, 95% CI 1.57–3.10) are strongly associated with the development of AKI in infants undergoing abdominal surgery (41).

#### Hypoalbuminemia

A secondary analysis of the AWAKEN, study found low serum albumin to be an independent factor of early and late neonatal AKI; for every 0.1 g/dl decrease in albumin, the odds of AKI development increased by 12% (48). Hypoalbuminemia is associated with severe illness and/or with poor nutrition, compounding the risk of AKI (49). Preoperative hypoalbuminemia was associated with postoperative AKI in Cui et al., however, analysis in multivariable models it did not maintain significance (10, 48).

#### High American society of anesthesiologist (ASA) Status

Anesthesiologists play a main role in preparations for abdominal surgery in infants. They evaluate and manage infants’ nociceptive system and stress response, respiratory status, fluid balance, blood loss, and temperature control (50). ASA physical status is routinely used to evaluate for risk of preoperative anesthesia (10). ASA score evaluates preanesthetic medical comorbidities, accounting for factors such as surgery type, frailty, and level of deconditioning (51). High ASA status (III and IV) contributes to AKI development after abdominal surgery in newborns (10).

### Intraoperative risk factors for AKI

#### Emergent vs. nonemergent surgery

There is conflicting data as to whether emergent abdominal surgeries are associated with increased risk of AKI (12). Slagle et al. demonstrated that the incidence of AKI was higher in a group of neonates with emergent abdominal procedures as compared to those infants who underwent elective procedures (64% vs. 30%, *p* < 0.01) (12). Conversely, Cui et al. did not find any significance in AKI rates between emergent and non-emergent surgery (10).

#### Hypotension and vasopressors

Hypotension and vasopressor requirement during abdominal surgery are associated with postoperative AKI. Hypotension leads to kidney hypoperfusion and thus AKI (28, 52, 53). Renal hypoperfusion can occur for many reasons, including excessive blood loss, sepsis with capillary leak, or inadequate fluid replacement (54) Lower intraoperative systolic blood pressure is associated with postoperative AKI (10). Infants requiring vasopressors during abdominal surgery had higher AKI rates as compared to infants who did not (50% vs. 30.8%, *p* = 0.05) (10). Infants with postoperative AKI had higher intraoperative fluid infusion rates as compared to infants who did not develop AKI (33 ml/kg/hour vs. 26.4 ml/kg/hour, *p* < 0.01) (10).

#### Blood loss

Blood loss with resultant anemia may be a risk factor for postoperative AKI. Wu et al. found that infants with intraoperative blood loss >10 ml/kg had higher rates of postoperative AKI as compared to infants with <10 ml/kg of blood loss (31.5% vs. 17% *p* = 0.036) (9). However, this association lost significance in multivariable models (9).

#### Surgical time

Data is conflicting as to whether surgical time is associated with risk of AKI. In a study evaluating both emergent and planned procedures, operative time greater than 120 min was found to more than double the odds of AKI in neonates after abdominal surgery (OR 2.7 95% CI 1.1–6.6) (9). However, a study analyzing only emergent intra-abdominal surgeries, found that the median operative time was significantly shorter in infants who developed postoperative AKI as compared to those who did not (10). This discrepancy may be due to the severity of illness of the infants undergoing emergency surgery and/or the differences in surgical procedures and techniques (10).

#### Intraoperative hypothermia

Infants are at risk for hypothermia in the operative room. Hypothermic neonates require more invasive interventions than neonates who maintain normothermia (55). Hypothermia may result in peripheral vascular contraction and decreased renal perfusion (11). Hypothermia throughout the intraoperative time is associated with increased rates of postoperative AKI (10, 11).

### Postoperative risk factors for AKI

#### Elevated serum lactate

Serum lactate is often monitored postoperatively to evaluate perfusion status. Wu et al. demonstrated that elevated serum lactate on the first postoperative day was predictive of postoperative AKI (14.9 mg/dl (11.1–19.3) vs. 16.3 mg/dl (12.1–20.4), *p* = 0.034) (9).

#### Nephrotoxic medications

As mentioned previously, nephrotoxins can compound or initiate kidney injury in infants. Slagle et al. demonstrated that AKI is seen in higher rates for infants exposed to nephrotoxic medications within 5 days of their operation (12).

### Prevention/mitigation of AKI

Caffeine is associated with a reduced incidence and severity of AKI (56). Methylxanthines like caffeine and theophylline may increase renal blood flow, enhance sodium excretion, and protect against oxidative stress (57). Aviles-Otero et al. studied the effects of preoperative caffeine exposure and found lower peak serum creatinine and absolute changes post-surgery as well as significantly reduced odds of postoperative AKI (55.5% vs. 92.6%, OR 0.10, 95% CI 0.02–0.44) (22).

### Early prediction of postoperative AKI

Diagnosis of AKI is dependent upon serum creatinine and urinary output changes (Supplementary Table S1). Elevations in serum creatinine can lag up to 48–72 h after kidney injury (40). Novel biomarkers, such as urine NGAL (uNGAL), can be used as an early marker of impending AKI (40). uNGAL is induced and secreted by distal tubular epithelial cells after injuries and has been studied in a number of infant populations implemented in many clinical settings, critically ill neonates and post-cardiac infants (58). Slagle et al.'s prospective observational study specifically evaluated the association of uNGAL after intrabdominal procedures and found elevated uNGAL 24 h after surgical intervention, to be predictive of postoperative AKI (AUC of 0.81, 95% CI, 0.89) (12). Early prediction of AKI can help risk stratify infants and implement targeted strategies to prevent and/or reduce the intensity of AKI.

## Clinical management

### Perioperative prevention of AKI

The management of newborns requiring abdominal therapy is complicated and requires a multidisciplinary team (55). The Enhanced Recovery After Surgery (ERAS) society, has proposed that the perioperative multidisciplinary team should utilize a checklist with a structured process and protocol to improve outcomes. These checklists can be adapted to consider the prevention and mitigation of postoperative AKI (55).

### Preoperative management

A full history, including antenatal complications, gestational age, delivery, and current clinical problems, should be obtained and discussed between the anesthesiologist, neonatologist and pediatric surgeon to optimize medical care for newborns prior to abdominal surgery (50). Preoperative labs, including CBC, coagulation factor, blood type, serum creatinine, bicarbonate electrolytes, liver function test, and baseline urinalysis, should be obtained. Nephrotoxin exposure should be minimized if feasible (neonatal ventilation, ECMO status, and fluid balance should be carefully evaluated) (50). Blood loss is one of the common complications in abdominal surgery in neonates and anemia and may potentiate AKI (55). To prevent severe effects of hypoxemia, it is recommended to maintain hemoglobin at or above 9 g/dl for term neonates older than one week and without oxygen requirement or, in those intubated with an oxygen requirement, at 11 g/dl for term neonates within the first week of life (55). Preoperative urine output and lowest serum creatinine should be recorded (55). To prevent surgical site infections and sepsis antibiotic prophylaxis should be given 60 min prior to skin incision (55).

### Intraoperative management

Temperature, hemodynamics, and urine output should be carefully monitored throughout the surgical procedure. The ERAS consensus recommends monitoring intraoperative core temperature continuously and taking preemptive measures to maintain normothermia at or above 36.5°C and prevent hypothermia (55). While there is conflicting data about the development of abdominal surgery related AKI after hypothermia, it is clear that careful management of fluid, electrolytes, and acid-base balance as well as prevention of hypoglycemia help to maintain tissue perfusion and prevent hypovolemia, fluid overload, and hypoglycemia/hyperglycemia. Blood transfusions, changes in ventilation, or drug use (pressors, diuretics, antibiotics, analgesia…) should be noted as these variables can often precipitate AKI events (50, 55).

### Postoperative management

The perioperative communication and care process can help to reduce adverse patient outcomes, including AKI. The postoperative handoff should include intraoperative events such as hypotension, excessive fluid administration, need for vasopressor support, and estimated blood loss. Neonates with the aforementioned risk factors should be monitored closely with strict intake and output, and daily weights. Labs, including CBC, electrolytes, and serum creatinine, should be monitored carefully. Nephrotoxic medications should be avoided if possible. If they must be used, ensure careful monitoring of troughs (e.g., Vancomycin/gentamicin) and consider screening daily creatinine values (50, 55).

## Discussion and future directions

Compared to the growing body of literature evaluating early detection and prevention of cardiac surgery associated AKI, there are few studies of AKI associated with abdominal surgery in infants. The available data is almost exclusively retrospective and limited to single-center experiences making it difficult to draw strong conclusions about predictors of postoperative AKI (59). What is clear is that AKI is not an uncommon phenomenon and is frequently associated with gastrointestinal pathologies and corrective surgery.

Given the frequency of AKI in this population, increased awareness of the associations of AKI with abdominal surgery among pediatric surgeons about may enhance patient outcomes. While some of the risk factors for AKI are common and previously reported in critically ill neonates and infants (i.e., sepsis, nephrotoxins, hypovolemia, anemia, gestational age, and mechanical ventilation), abdominal surgery has unique features that predispose and/or exacerbate AKI such as higher risk of gut bacterial translocation and abdominal compartment syndrome (3, 58). Cardiac surgeons are aware of AKI as an outcome of pediatric cardiac surgery due to regulatory Quality Improvement (QI) (60, 61). It may be effective for similar metrics to be developed for general surgeons in order to ensure that kidney health is a goal of postoperative care.

In addition to increased AKI recognition among general surgeons, prospective studies of early biomarkers of AKI, in large multicenter cohorts of infants would be crucial to evaluate risk and prompt diagnosis. It is exciting that Slagle et al.'s single-center prospective study demonstrated that the percent change of urinary NGAL pre and post operatively was a sensitive early predictor of AKI development. Further evaluation regarding the use of biomarkers such as urinary NGAL to implement nephroprotective clinical practices (i.e., avoidance of nephrotoxic medications, careful fluid balance etc.) and evaluate if mitigative strategies can reduce or prevent postoperative AKI.

## Conclusion

Infants with gastrointestinal pathology are at increased risk of postoperative AKI due to preoperative, intraoperative, and postoperative exposures. Identification of associated risk factors, avoidance of nephrotoxic medications, careful fluid balance, and early detection is imperative to improve the care of neonates with abdominal surgery. Further prospective studies are needed to test interventions pre-, intra-, and post-abdominal surgery that prevent and/or mitigate postoperative AKI.
