# Supplementary material for: Acute kidney injury post-abdominal surgery in infants: implications for prevention and management
Source: Front Pediatr. 2023 Apr 21;11:1162863. doi: 10.3389/fped.2023.1162863 (PMC10160454; doi:10.3389/fped.2023.1162863)
Supplement: Supplementary file 1 [file Datasheet1.pdf]

**Supplementary Table 1**. Kidney Disease Improving Global Outcomes (KDIGO) Modified for Neonates Creatine and Urine Output Criteria*

| Stage | Serum creatinine (SCr) | Urine output over 24 hours |
|---|---|---|
| 0 | No change or increase < 0·3 mg/dL | > 1 mL/kg/hour |
| 1 | Increase ≥ 0.3 mg/dL within 48 hours or<br>Increase ≥1.5–1.9 × reference SCr[a] within 7 days | >0.5 and ≤ 1 mL/kg/hour |
| 2 | Increase ≥ 2.0- 2.9 × reference SCr[a] | >0.3 and ≤0.5 mL/kg/hour |
| 3 | Increase ≥ 3 × reference SCr[b] or<br>SCr ≥ 2.5 mg/dL[b] or<br>Receipt of dialysis | ≤ 0.3 mL/kg/hour |

* Adapted from Jetton, J. G et al. (1)

[a] Reference SCr is the lowest prior SCr measurement
[b] SCr of 2.5 mg/dl in neonates suggests a GFR < 10 ml/min/1.73m$^2$

Supplementary Table 2. Diagnoses associated with postoperative AKI classified by pathophysiologic etiology *

| Study | (1) Peritonitis | (2) Intestinal Obstruction | (3) Abdominal Wall Anomalies and Compartment Syndrome |
|---|---|---|---|
| Wu, Y., et al. (2) | • Spontaneous intestinal perforation<br>• Necrotizing enterocolitis | • Duodenal atresia and stenosis<br>• Jejunoileal atresia and stenosis<br>• Meconium ileus<br>• Hirschsprung's disease<br>• Anorectal malformation | • Congenital defects of the abdominal wall |
| Slagle CL et al. (3) | • Acute necrotizing enterocolitis<br>• Intestinal perforation | • Bowel obstruction or atresia<br>• Short bowel syndrome/ Intestinal failure | • Omphalocele<br>• Gastroschisis |
| Cui Y et al. (4) | • Gastric perforation<br>• Intestinal perforation<br>• Necrotizing enterocolitis<br>• Appendicitis | • Intestinal atresia<br>• Intestinal stenosis<br>• Ileus<br>• Intestinal volvulus<br>• Pyloric hypertrophy<br>• Intestinal malrotation<br>• Congenital anorectal malformation | • Gastroschisis<br>• Umbilical bulge |
| Cui Y, Cao R , and Deng L (5) | • Gastric perforation<br>• Appendicitis | • Intestinal volvulus<br>• Pyloric stenosis<br>• Anorectal malformation<br>• Anomalies of rotation | • Umbilical bulge |
| Yum SK et al. (6) | • Necrotizing enterocolitis<br>• Bowel perforation<br>• Hemorrhagic necrosis/Inflammation | • Meconium-related disease<br>• Intussusception | • Congenital anomaly |

* 6 studies which number of surgical procedures were recorded for completed abdominal operation only. To better understand the pathologies, above studies were categorized into 3 categories based on Rocha G et al. (7). Any pathology or surgical intervention not specified or involved thoracic were excluded.

**References**


1. Jetton JG, Boohaker LJ, Sethi SK, Wazir S, Rohatgi S, Soranno DE, et al. Incidence and outcomes of neonatal acute kidney injury (AWAKEN): a multicentre, multinational, observational cohort study. The lancet child & adolescent health. 2017;1(3):184-94.

2. Wu Y, Hua X, Yang G, Xiang B, Jiang X. Incidence, risk factors, and outcomes of acute kidney injury in neonates after surgical procedures. Pediatric Nephrology. 2020;35(7):1341-6.

3. Slagle CL, Goldstein SL, Gavigan HW, Rowe JA, Krallman KA, Kaplan HC, et al. Association between elevated urine neutrophil gelatinase-associated lipocalin and postoperative acute kidney injury in neonates. The Journal of Pediatrics. 2021;238:193-201. e2.

4. Cui Y, Fang X, Li J, Deng L. Evaluation of neonatal acute kidney injury (AKI) after emergency gastrointestinal surgery. Asian Journal of Surgery. 2022.

5. Cui Y, Cao R, Deng L. Inadvertent hypothermia and acute kidney injury (AKI) in neonates undergoing gastrointestinal surgeries: a retrospective study. Journal of Perinatology. 2022;42(2):247-53.

6. Yum SK, Seo YM, Youn YA, Sung IK. Preoperative metabolic acidosis and acute kidney injury after open laparotomy in the neonatal intensive care unit. Pediatrics International. 2019;61(10):994-1000.

7. Rocha G, Costa C, Correia-Pinto J, Monteiro J, Guimarães H. The acute abdomen in the newborn. Acta Médica Portuguesa. 2009;22(5):559-66.
